# Leaf Development in *Medicago truncatula*

**DOI:** 10.3390/genes13071203

**Published:** 2022-07-05

**Authors:** Liren Du, Samuel Adkins, Mingli Xu

**Affiliations:** Department of Biological Sciences, University of South Carolina, Columbia, SC 29208, USA; liren@email.sc.edu (L.D.); swadkins@email.sc.edu (S.A.)

**Keywords:** leaf development, morphogenesis, *Medicago*, leaflet number, leaf size, leaf shape

## Abstract

Forage yield is largely dependent on leaf development, during which the number of leaves, leaflets, leaf size, and shape are determined. In this mini-review, we briefly summarize recent studies of leaf development in *Medicago truncatula*, a model plant for legumes, with a focus on factors that could affect biomass of leaves. These include: floral development and related genes, lateral organ boundary genes, auxin biosynthesis, transportation and signaling genes, and *WOX* related genes.

## 1. Introduction

Leaves are the primary organ for photosynthesis and provide energy to both plants and primary consumers. Although fruits and seeds produced by plants are more commonly thought of as food, many animals consume plant leaves as a source of energy and nutrients. The forage leaves that feed many agriculturally important herd animals are of particular interest. Forage yield is largely dependent on the number, size, and shape of forage leaves; therefore, plant leaf development has a great effect on proper forage yield results. *Medicago truncatula* (*M. truncatula*), an annual forage legume that is closely related to the world’s most economically important forage legume alfalfa (*Medicago sativa*), has been employed as a model plant for legumes because of its simpler genetics and easier growth habit [1]. In *M. truncatula*, normal leaf development procedures are divided into three stages. Leaves are first initiated as rod-like primordia from the flanks of the shoot apical meristems (SAM) before undergoing primary morphogenesis and secondary morphogenesis [2]. During primary morphogenesis—otherwise known as morphogenesis—the lamina is initiated and leaf marginal structures such as leaflets, lobes, and serrations are formed. During secondary morphogenesis—also referred to as differentiation—leaf tissues grow, differentiate, and mature, and the final leaf shape is formed [2]. Both *M. truncatula* and *Medicago sativa* undergo these three stages of leaf development and develop compound leaves in which more than one leaflet is attached to their petiole. As *M. truncatula* is a well-studied model plant for legumes, in this mini-review we will discuss factors that may affect the number of leaflets per petiole, and size and shape of leaves during the three leaf developmental stages of *M. truncatula*.

## 2. Regulation of the Number of Leaflets per Leaf

Simple and compound leaves are two basic forms of leaves in higher plants. Simple leaves have a single undivided blade per petiole, while compound leaves have multiple blade units, called leaflets, per petiole [2]. Compound leaves may have an evolutionary advantage, as they have more blades for photosynthesis and herbivory survival; therefore, mechanisms that determine simple and compound leaf variation are some of the major factors that determines number of leaflets per petiole, while other factors may also promote the presence of more or less leaflets per petiole.

### 2.1. KNOX Genes

**Figure 1:**
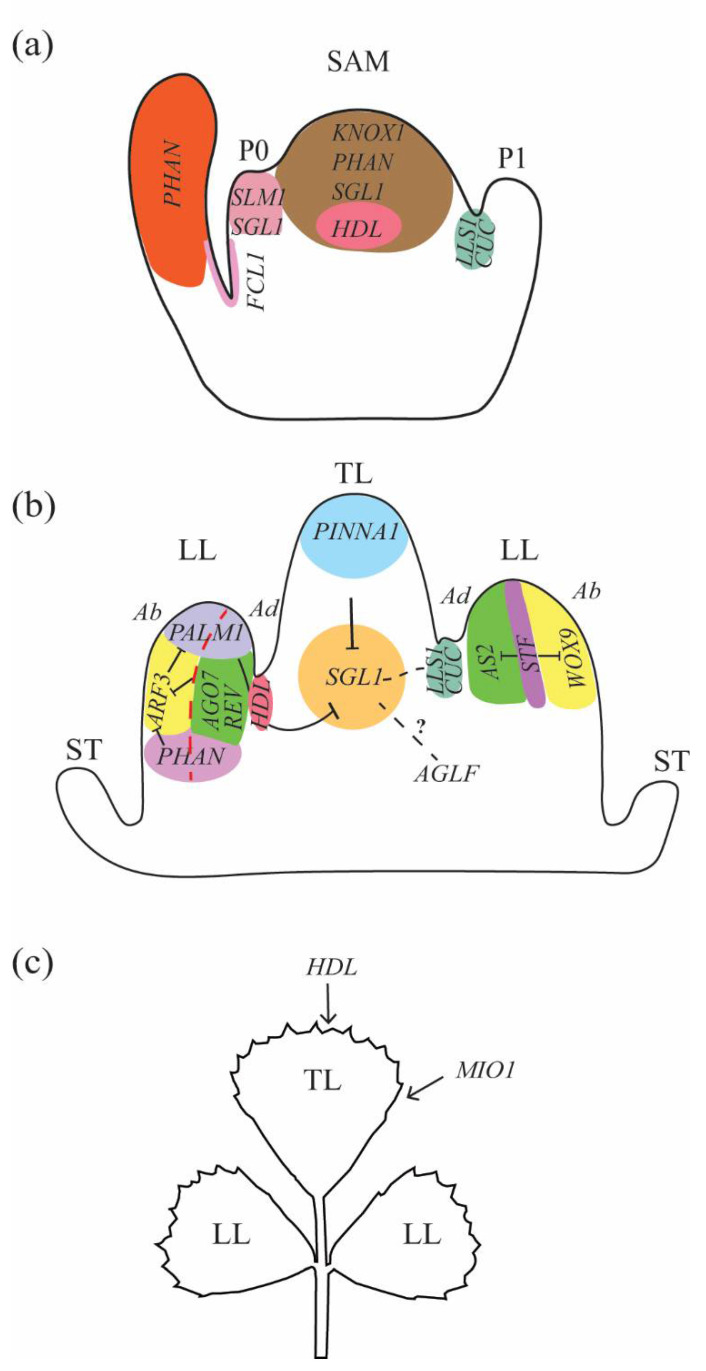
(**a**) Gene networks controlling compound leaf development of *Medicago truncatula* at the initiation stage. *KNOX I* genes are expressed in SAM and down regulated in leaf primordia. *PHAN* and *SGL1* are expressed at both SAM and early-stage leaf primordia during development. *HDL* is expressed in the organization center of the SAM. *SLM1* and *SGL1* are expressed at early-stage leaf primordia. *FCL1*, *LLS1*, and *CUC* genes are expressed at the boundary between SAM and leaf primordia. (**b**) Gene networks controlling the compound leaf development of *Medicago truncatula* at the morphogenesis stage. *PALM1* is expressed at lateral leaflet primordia and *PINNA1* is expressed at the distal region of terminal leaflet primordia. *SGL1* is restricted to the basal region of the terminal leaflet primordia by PALM1 and PINNA1. *LLS1* and *CUC* are expressed at the boundary between terminal leaflet primordia and lateral leaflet primordia. *HDL* is expressed at the axis between the terminal leaflet primordia and lateral leaflet primordia to suppress extra leaflet primordia outgrowth. *REV* is expressed at the adaxial portion of leaf primordia to regulate auxin-related genes. AGO7 and PHAN repress *ARF3*, which represses *PALM1* during leaf morphogenesis. *STF* is expressed at the boundary between the adaxial–abaxial domain to restrict the expression of *AS2* and *WOX9*. (**c**) Genes control lamina size and shape at the differentiation stage. HDL promotes margin serration and lamina length/width ratio, while MIO1 promote cell proliferation to promote lamina size. TL: terminal leaflet. LL: lateral leaflets. ST: stipules. Ab: Abaxial. Ad: Adaxial.

In *Arabidopsis thaliana*, a plant with simple leaves, the class I *KNOTTED1-LIKE HOMEOBOX* (*KNOXI*) genes, including *SHOOTMERISTEMLESS* (*STM*), *KNOTTED-LIKE FROM ARABIDOPSIS THALIANA 1* (*KNAT1*), *KNAT2*, and *KNAT6*, are expressed in the SAM and are excluded from the leaves, while MYB domain transcription factor *ASYMMTRIC LEAVES 1* (*AS1*), LOB domain transcription factor *AS2*, and BTB-POZ domain transcription cofactors *BLADE-ON-PETIOLE 1* (*BOP1*) and *2* are expressed in the leaves and not in the SAM [3,4,5,6,7]. Loss-of-function *as1* and *as2* single mutants and *bop1 bop2* double mutants caused ectopic growth on their petioles, which resemble the compound leaf form [5,6,7,8,9,10]. Molecular and genetic analysis indicates that meristematic *KNOXI* genes *STM*, *KNAT1*, *KNAT2*, and *KNAT6* are misexpressed in *as1*, *as2*, and *bop1 bop2* leaves, and the inactivation of these *KNOXI* genes in these mutants resulted in reduced growth of their petioles. These results indicate that simple and compound leaf variation might be caused by different expressions of *KNOXI* genes in leaves [7,9,10,11,12,13]. This hypothesis was supported by studies in *Cardamine hirsuta*, where the expression of *STM* and *KNAT1* is reactivated in its leaf primordia, which gives rise to compound leaves [14]. Further analysis showed that the ectopic expression of *STM* or *KNATI* in *Cardamine* resulted in more leaflets per petiole and reduced activities of STM resulted in less leaflets per petiole, which supports the hypothesis that controlling the expression of *KNOXI* genes in leaves could induce simple and compound leaf variation [14]. Surprisingly, studies carried out on legume models do not fully support this hypothesis. Both peas and soybeans produce compound leaves, but *KNOXI* genes behave differently. *KNOXI* genes are expressed in soybean leaf primordia, and an overexpression of such genes results in more leaflets per petiole in alfalfa [15]. However, these genes are not expressed in pea leaf primordia, nor in *M. truncatula*, a close relative to soybean that also has compound leaves [15,16,17,18] (Figure 1a). *PHANTASTICA* (*PHAN*) from *Antirrhinum majus* is an ortholog of *AS1* from *Arabidopsis* and both promote abaxial–adaxial polarity in leaf development [19] (Figure 1a). *KNOXI* genes are reactivated in *Arabidopsis as1* leaves [3,5]; however, *KNOX1*, *KNOX2*, and *KNOX6* are not reactivated in the *M. truncatula phan* mutant, though *KNOX7* is reactivated in *M. truncatula phan* petiole. The *phan* mutant did not show any changes in the complexity of leaves [17,18]. These studies suggest that the mechanisms for controlling the number of leaflets per petiole in *M. truncatula* differ from other compound leaf species.

### 2.2. Floral Development and Related Genes

Wild type *M. truncatula* has three leaflets per petiole, which was reduced to one by mutations in *SINGLE LEAFLET1* (*SGL1*), suggesting that the roles of *KNOXI* genes in *Cardamine* may be replaced by *SGL1* during evolution [20]. *SGL1* encodes a *LEAFY* (*LFY*) ortholog from *Arabidopsis*, which is a master regulator for floral organogenesis. *sgl1* also has defects in floral organogenesis, like *lfy* mutants in *Arabidopsis* [20]. *SGL1* is detected at SAM and leaf primordia (Figure 1a,b). Close examination of leaf development in *sgl1* mutants suggest that SGL1 promotes lateral lamina primordia—a hallmark of morphogenesis—and leads to the outgrowth of lateral leaflet on the petiole; conversely, the C2H2 zinc finger transcription factor PALMATE-LIKE PENTAFOLIATA1 (PALM1) functions to suppress lateral leaflet outgrowth in *M. truncatula* [21]. *PALM1* is expressed in the lateral leaflet primordia to keep the expression of *SGL1* at moderate levels and prevent extra leaflet outgrowth (Figure 1b). Mutation in *PALM1* resulted in the outgrowth of two extra leaflets, creating a total of five leaflets instead of three [21,22]. Similar to *PALM1*, mutations in a BEL1-like homeodomain protein *PINNATE-LIKE PENTAFOLIATA1* (*PINNA1*) also resulted in 5 leaflets per petiole. *PINNA1* is expressed at the distal of the leaf primordia to restrict the expression of *SGL1* at the base of leaf primordia (Figure 1b). *pinna1* mutants have two extra leaflets below the terminal leaflet, which differs from the *palm1* mutant which has two extra leaflets below the lateral leaflets. Mutation in *PALM1* and *PINNA1* simultaneously resulted in complicated leaves, suggesting that PALM1 and PINNA1 function synergistically in modulating the number of leaflets per petiole [22]. Another possible floral factor that influences leaf development is a gene similar to *AGAMOUS* (*AG*) in *M. truncatula. AG* is a C-class function gene in *Arabidopsis* that controls the stamen and carpel identity in *Arabidopsis* flower development. *AGAMOUS-LIKE FLOWER* (*AGLF*) in *M. truncatula* controls both flower development and leaf development [23,24] (Figure 1b). In *aglf-1* mutants, the petiole length is reduced, causing clustered leaflets in the mutant [24]. It is unknown if AGLF interacts with SGL1, PALM1, PINNA1, or other factors in regulating the complexity of *M. truncatula* leaves.

### 2.3. Lateral Organ Boundary Genes

Deep leaf margin serration can cause a blade to divide, resulting in multiple leaflet-like structures at the distal of leaf [25]. The lateral organ boundary gene *CUP-SHAPED COTYLEDON3* (*CUC3*) is expressed at organ boundaries in *Arabidopsis* and at the boundary of leaflet primordia in several compound leaf species [26,27]. Ectopic expression of *CUC* genes in *Arabidopsis* resulted in highly serrated leaf margins [26], and reducing expression levels of *CUC* in *Cardamine* resulted in smoother leaf margins and less leaflets [27]. These findings suggest that multiple leaflets could be caused from deep leaf margin serration. In *M. truncatula*, *CUC2*/*NAM* is expressed at the boundary between the cotyledons and SAM, and at the boundary between leaflets (Figure 1a,b); consequently, mutation in *MtCUC2*/*NAM* resulted in fused cotyledons and fused leaflets, mimicking simple leaves [28,29]. Fused leaflets are also observed in the class M *KNOX* gene mutant *fused compound leaf 1* (*fcl1*) where *FCL1* is expressed at incipient leaf primordia and at the boundary between SAM and leaf primordia in *Medicago* [16] (Figure 1a). *FCL1* encodes a *KNOX* gene that contains the *KNOX1* and *KNOX2* domains and lacks the homeodomain, resulting in a distinct expression pattern and function from *KNOXI* genes [16]. The *sgl1 nam* double mutant produced one leaflet, the same phenotype as the *sgl1* single mutant, suggesting that *sgl1* is epistatic to *nam* in regulating number of leaflets [29]. These results suggest that *SGL1* and organ boundary genes have different roles in leaf development even though mutations in both resulted in simple leaves. SGL1 promotes lateral leaflet primordia formation while organ boundary genes promote leaflets separation during compound leaf development [16].

### 2.4. Auxin

The phytohormone auxin has many roles in plant development, including leaf, flower, and root development [30,31]. Simple leaf species *Arabidopsis* has a smooth petiole during morphogenesis, and there is no auxin maximum in the petiole; conversely, auxin maximum was observed in the petiole of *Cardamine* during leaf morphogenesis and mutations in the polar auxin transport efflux carrier *PINFORMED1* (*PIN1*) resulted in less leaflets, suggesting that the formation of multiple leaflets during morphogenesis requires auxin accumulation in the petiole [32]. Several lines of evidence suggest that auxin is also required for multiple leaflet outgrowth in *M. truncatula*. *SMOOTH LEAF MARGIN1* (*SLM1*) is an ortholog of the auxin efflux carrier *PIN1* from *Arabidopsis* that is expressed at the leaf primordia and leaf margins of *M. truncatula* (Figure 1a,c). Mutation in *SLM1* resulted in smooth leaf margins and more than 3 leaflets per petiole, and the *slm1 sgl1* double mutant has more leaflets than *sgl1* and less leaflets than *slm1*, suggesting antagonistic interactions between SLM1 and SGL1 [33]. *LATERAL LEAFLET SUPPRESSION1* (*LLS1*) in *Medicago* is an ortholog to auxin biosynthetic enzyme *YUCCA1* from *Arabidopsis*. *LLS1* is expressed in early-stage leaf primordia and at the basal regions between the terminal leaflet and lateral leaflet primordia (Figure 1b). Mutations in *LLS1* resulted in suppression of the lateral leaflet outgrowth, like *SGL1* [34]. *lls1* could not change the phenotypes in *sgl1* but reduced the number of leaflets in *palm1*, suggesting that LLS1 functions synergically to SGL1 and antagonistically to PALM1 [34]. AUXIN RESPONSE FACTORS (ARF) function downstream in auxin signaling and mediate auxin effects [31]. Yeast one-hybrid screening revealed that MtARF3 physically binds to *PALM1* promoter, suggesting that auxin is associated with PALM1. *AGO7* is expressed at the adaxial portion of leaf primordia, and *PHAN* is expressed in both the adaxial and abaxial portion of leaf primordia [35]. Together, PHAN and AGO7 restrict *ARF3* at the abaxial portion of the leaf primordia. Simultaneous mutations in *AGO7* and *PHAN*, two repressors for ARF3 (Figure 1b), resulted in downregulation of *PALM1* and 5 leaflets per petiole, resembling the *palml1* mutant [35]. Auxin homeostasis, number of leaflets per petiole, and leaflet shape are also regulated by HD-ZIPIII gene *REVOLUTA* (*MtREV*), which is specifically expressed in the adaxial portion of leaf [36] (Figure 1b). These studies suggest that leaf polarity regulators are key regulators for auxin signalling and response, and likely regulate leaflet number through auxin. Finally, *HEADLESS* (*HDL*), encoding a *WUSCHEL* (*WUS*) ortholog from *Arabidopsis*, has roles in forming shoot apical meristems, leaf margin serration, and controlling the number of leaflets per petiole, likely through its regulation on auxin efflux carrier *SLM1*/*PIN1* [37,38].

## 3. Regulation of the Size and Shape of the Leaf

The size and shape of leaves also determines the biomass of leaves and therefore affects forage yields. In *Arabidopsis,* WUS controls meristem activity in the shoot apical meristems and flowers, while *WUS*-*related homeobox* (*WOX*) genes have been found to regulate cell proliferation in leaves in both monocots and dicots. *WOX1* and *WOX3*/*PRESSED FLOWER* (*PRS*) are expressed in *Arabidopsis* leaf primordia at the adaxial and abaxial boundary while the *wox1 prs* double mutant has narrower lamina than the wild type, indicating the roles of WOX genes in promoting cell proliferation along the mediolateral axis in leaves [39]. As with *WOX1* and *WOX3*, mutations in the maize *WOX3* ortholog *NARROW SHEATH1* (*NS1*) and *NS2*, also resulted in exceptionally narrow leaves [40]. Likewise, the *WOX1* ortholog in *M. truncatula STENOFOLIA* (*STF*) is expressed at the boundary between the adaxial–abaxial domain and has important roles in lamina expansion by promoting cell proliferation in leaves [41] (Figure 1b). Mutations in *STF* resulted in narrow and long leaves (in *Nicotiana sylvestris*) or leaflets (in *M. truncatula*), while overexpression of *STF* resulted in wider lamina and greater biomass in *Brachypodium* and switchgrass [42]. STF also interacts with TOPLESS (TPL) to promote blade expansion through the *MtAS2* dependent pathway and cytokinin pathways [42,43]. Additionally, ANGUSTIFOLIA3 (AN3) is a transcription coactivator and LEUNIG (LUG) is a transcription corepressor with both having roles in leaf development [44,45]. Studies showed that STF interacts with both AN3 and LUG physically and LUG may function as transcription coactivator to regulate lamina expansion similar to AN3 [46]. In leaf blade expansion, *STF* also directly represses *WOX9*, another member of the *WOX* gene family that is expressed at the leaf’s abaxial domain [47] (Figure 1b). *HDL* is an *Arabidopsis WUS* ortholog and is expressed at leaf margin serrations. Mutation in *HDL* resulted in shorter blades and higher blade width/length ratio [38] (Figure 1c). This suggests that *HDL* is involved in promoting lamina shape along the proximal–distal axis, but the mechanism remains unknown.

Although *WOX* and related genes were found to control leaflet shape, an F-box protein MINI ORGAN1 (MIO1)/SMALL LEAF AND BUSHY1 (SLB1) was discovered to regulate leaflet size in *Medicago* (Figure 1c). The morphology of leaflets and flowers was not changed in loss-of-function *mio1*, but the size of leaflets and flowers were reduced, which resulted in smaller leaflets and flowers; conversely, overexpression of *MIO1* resulted in larger leaflets [48]. Mutations in *STERILE APETALA (SAP)*/*SUPPRESSOR OF DA1* (*SOD3*), the *Arabidopsis* ortholog of *MIO1*/*SLB1*, also resulted in smaller leaves, and ectopic expression of *MIO1* in *Arabidopsis* (*35S::MIO1*) could rescue the small leaves in *sod3*, indicating conserved roles of *MIO1*/*SOD3* in plants [48]; however, *MIO1* is expressed in leaf primordia and localized in nucleus to regulate cell division, not cell expansion, to regulate leaflet size [48]. The targets of MIO1 have yet to be determined, as is the mechanism of regulation.

## 4. Challenges and Perspectives

Forage yield is determined largely by leaf development in plants. In this mini-review, we summarized leaf development factors that could potentially affect overall leaf yield in the forage model plant *M. truncatula*. Number of leaflets per petiole is a critical influencer on the overall production of leaves, and several factors are involved in regulating the number of leaflets per petiole. These include floral development orthologs *SGL1* and *AGLF*, and factors that interact with them (such as *PALM1*, *PINNA1*); Auxin and auxin signaling related genes; organ boundary gene *CUC2*/*NAM*, and *FCL1*. The shape and size of leaflets may also affect leaf yield. The *WOX* gene family seems to have essential roles in promoting blade expansion and STF (WOX1) represses the leaf adaxial–abaxial polarity genes *AS2* and *WOX9* to promote cell division along the medial–lateral axis. MIO1 promotes leaflet size by promoting cell division activities in leaf primordia; however, the mechanisms of how MIO1 acts remain to be determined. In *Arabidopsis*, a series of genes have been found to regulate final leaf size by regulating cell proliferation, including GROWTHREGULATING FACTOR1 (GRF1), GRF2, and GRF5, ANGUSTIFOLIA3 (AN3)/GRF-INTERACTING FACTOR, KLUH, AVP1, JAW, BRI1, and GA [49,50,51,52]. In maize, leaf size is regulated by ARGONAUTE, MADS box, SQUAMOSA PROMOTER-BINDING PROTEIN (SBP), GRF, bZIP and TCP transcription factors, and epigenetic factors [53]. It will be of particular interest to know if these life size regulators in *Arabidopsis* and maize function in *Medicago*. A third factor possibly affecting leaf yield is the rate of leaf initiation, or the plastochron length. Faster leaf initiation or smaller plastochron length could result in more leaves produced and higher forage yield during vegetative development. The microRNA *miR156* and its targets *SQUAMOSA PROMOTER BINDING PROTEIN-LIKE* (*SPL*) genes are involved in regulating plastochron length and leaf shape in *Arabidopsis* [54,55]. Several lines of evidence suggest that the miR156-SPL module could be a promising tool for improving forage yield. The leaf complexity in *Cardamine* is age-dependent and is correlated to expression levels of the floral repressor *FLOWERING LOCUS C* (*FLC*) [56]; it is also likely that effects of FLC are mediated through SPL genes [56]. It is reported in *Medicago sativa* (alfalfa) that plants overexpressing *miR156* show a bushy phenotype and enhanced biomass yield [57]; however, it is unknown if these phenotypes are related to plastochron length. Mutations in *MtSPL8* and *MtSPL13* caused more branching and increased biomass in *M. truncatula* [58,59], and mutations in *MtSPL4* caused more leaflets per petiole. Future research may determine if the miR156-SPL module also regulates plastochron length in addition to regulating branching and number of leaflets per petiole in *Medicago*, adding an additional layer to increase forage yield. In *Cardamine*, a ribosome-associated protein SIMPLE LEAF 3 (SIL3) was reported to affect both leaflet number and plastonchron length through regulating auxin accumulation and *KNOXI* genes [60]. Ribosome encoding proteins PIGGYBACK1 (PGY1), PGY2, and PGY3 are involved in establishing leaf adaxial–abaxial polarity in *Arabidopsis* [61]. It will be interesting to understand how the ribosome related proteins function in *Medicago*, particularly if they regulate leaflet number or leaf size that could affect forage yield.
